# Recombination Blurs Phylogenetic Groups Routine Assignment in *Escherichia coli*: Setting the Record Straight

**DOI:** 10.1371/journal.pone.0105395

**Published:** 2014-08-19

**Authors:** María-Carmen Turrientes, José-María González-Alba, Rosa del Campo, María-Rosario Baquero, Rafael Cantón, Fernando Baquero, Juan Carlos Galán

**Affiliations:** 1 Servicio de Microbiología, Hospital Universitario Ramón y Cajal, Instituto Ramón y Cajal de Investigación Sanitaria (IRYCIS), Madrid, Spain; 2 CIBER en Epidemiología y Salud Pública (CIBERESP), Madrid, Spain; 3 Red Española para la Investigación en Enfermedades Infecciosas (REIPI), Madrid, Spain; 4 Universidad Alfonso X El Sabio, Villanueva de la Cañada, Madrid, Spain; State Key Laboratory of Pathogen and Biosecurity, Beijing Institute of Microbiology and Epidemiology, China

## Abstract

The characterization of population structures plays a main role for understanding outbreaks and the dynamics of bacterial spreading. In *Escherichia coli*, the widely used combination of multiplex-PCR scheme together with goeBURST has some limitations. The purpose of this study is to show that the combination of different phylogenetic approaches based on concatenated sequences of MLST genes results in a more precise assignment of *E. coli* phylogenetic groups, complete understanding of population structure and reconstruction of ancestral clones. A collection of 80 *Escherichia coli* strains of different origins was analyzed following the Clermont and Doumith's multiplex-PCR schemes. Doumith's multiplex-PCR showed only 1.7% of misassignment, whereas Clermont's-2000 protocol reached 14.0%, although the discrepancies reached 30% and 38.7% respectively when recombinant C, F and E phylogroups were considered. Therefore, correct phylogroup attribution is highly variable and depends on the clonal composition of the sample. As far as population structure of these *E. coli* strains, including 48 *E. coli* genomes from GenBank, goeBURST provides a quite dispersed population structure; whereas NeighborNet approach reveals a complex population structure. MLST-based eBURST can infer different founder genotypes, for instance ST23/ST88 could be detected as the founder genotypes for STC23; however, phylogenetic reconstructions might suggest ST410 as the ancestor clone and several evolutionary trajectories with different founders. To improve our routine understanding of *E. coli* molecular epidemiology, we propose a strategy based on three successive steps; first, to discriminate three main groups A/B1/C, D/F/E and B2 following Doumith's protocol; second, visualization of population structure based on MLST genes according to goeBURST, using NeighborNet to establish more complex relationships among STs; and third, to perform, a cost-free characterization of evolutionary trajectories in variants emerging along the clonal expansion using parsimony methods of phylogenetic analysis.

## Introduction

One of the most promising fields in bacterial molecular epidemiology is the characterization of the dynamics of changes in bacterial populations across spatial and/or temporal frames. Pulse-field gel electrophoresis techniques are useful for determining local clonal outbreaks but the current reconstructions of population structures in *Escherichia coli* are based on the combined results of two different analytical approaches. The first identifies macro-evolutionary events based on the assignment of particular strains to phylogenetic lineages; these macro-evolutionary events reflect bacterial “speciation-like” processes along large scales of time and space [1]. The second approach detects micro-evolutionary events, reflecting recent variations involved in local adaptations. These detections are based in practice on the identification of allelic variants in short fragments of sequence corresponding to seven selected housekeeping genes (MultiLocus Sequence Typing (MLST) patterns) [2], [3]. The combination of micro-and macro-evolutionary analyses has obvious applications for the understanding of the recent local spread of particular clones and can be applied to infer the evolution when the nucleotide background of the lineages is defined.

The most common approach for assigning phylogenetic lineages of *E. coli* is the simple and rapid multiplex PCR technique, used in microbial epidemiology laboratories for routine assignment of *E. coli* phylogroups [4], [5]. With this basic multiplex PCR-based method, *E. coli* strains can be classified into four phylogenetic groups (A, B1, B2, D) based on the presence or absence of the genes: *chuA*, and *yjaA* and the DNA fragment TSP4.C2, encoding a putative lipase esterase gene [6]. This multiplex PCR strategy is still widely used due to its simplicity and low price although the results present several limitations. In fact phylogenetic analyses of data from both whole genome sequencing and MLST have established the existence of at least seven *E. coli* phylogroups *sensu-stricto* [7]–[12].

The precise characterization of bacterial strains based on alleles and allelic profiles inferred for MLST (micro-evolutionary events) results in patterns easily comparable between different labs around the world. This method allows comparisons among strains from different places facilitating hospital-based, intra-country or international epidemiological studies. Analyzing all allelic profiles, eBURST and global optimal eBURST (goeBURST) algorithms are able to identify simple patterns of genetic relations in the population structure buffering the effect of recombination [13], [14]. The goeBURST representation enables allocation of particular strains within clones or clonal complexes eventually associated with different ecological ensembles [14]. This type of representation is easy to obtain, has a good discriminatory power, especially when applied to populations with a high clonality followed along a short period of time, and founder genotypes are faithfully detected.

The main limitations of goeBURST representation is that it cannot be used for inferring phylogenetic reconstructions *sensu-stricto* [13], and does not distinguish whether the allelic profiles are the result of point mutations (single or multiple) or recombination event(s). In bacterial species, such as *E. coli*, with relatively high recombination rates [15]–[17] goeBURST could lead to interpretative mistakes. Both sequencing technologies and evolutionary bioinformatics software are now increasingly available, and their integration provides new possibilities for increasing our understanding of evolutionary relationships in population structures. For instance, creation of phylogenetic reconstructions to determine the ancestral genotype, or phylodynamic analysis for following changes in population structure during an clonal expansion, or phylogeographical methods for defining the dispersion routes.

In this work, the combination of the widely used protocols based on multiplex PCR techniques and MLST-based goeBURST algorithm was compared to phylogenetic analysis using the same nucleotide information of *E. coli* MLST genes. Although this phylogenetic approach can significantly improve our understanding of evolutionary relationships, a wide surveillance of *E. coli* phylogroups characterization is still dependent on the availability of simple methods, such as the multiplex PCR mentioned above. Therefore, we would like to suggest a stepwise approach supplementing these widely used methods with accurate phylogenetic analysis, without additional economic cost.

## Material and Methods

### Ethics Statement

*E. coli* strains isolated from sheep belong to a bacterial collection deposited in the Veterinary Hospital of Alfonso X el Sabio University. The samples were recovered from rectum in natural flocks of sheep, using routine protocols. This veterinarian activity was included in the routine surveillance protocol. The veterinarians did not use drugs, special procedures and no sheep were sacrificed. This part of the work was supervised by Maria-Rosario Baquero (co-author in this manuscript).

The strains obtained from wastewater belonging to VISAVET collection (Centre for Veterinary Health Surveillance) were donated by Lucas Domínguez (Head of Department of VISAVET). We have an agreement about the use of strains from VISAVET Centre.

### Bacterial strains and genomic DNA extraction

Eighty *E. coli* strains from different origins and 48 *E. coli* complete genomes downloaded from GenBank (Table S1 in File S1) were included in the collection analysed in this study. Forty-one clinical strains of 80 strains recovered from extra-intestinal infections (bacteraemia and urinary infections) and 17/80 commensal *E. coli* strains obtained from healthy volunteers were collected at Ramón y Cajal University Hospital in Madrid, Spain. This collection was supplemented with five strains isolated from sheep faecal samples and, 17 strains isolated from wastewater. All 80 *E. coli* strains were incubated in 5 ml Luria Bertani broth in a shaking incubator for 24 h at 37°C. Genomic DNA was obtained using 1 mL of overnight bacterial growth, following manual extraction protocols as recommended by the manufacturers (QIAamp DNA Mini Kit. QIAGEN GmbH, Hilden, Germany).

### Determination of phylogroups, and sequencing of MLST genes for population analysis and evolutionary reconstructions

Initial determination of the phylogenetic groups in our *E. coli* strain collection was performed following two different, common multiplex PCR protocols based on the presence/absence of three genes: *chuA*, *yjaA* and *TSPE4.C2* [4], [5]. Primers and PCR conditions for the seven housekeeping genes commonly used in *E. coli* MLST schemes (*adk, fumC, icd, mdh, purA, recA* and *gyrB*) were obtained from databases at the Warwick University website (http://mlst.warwick.ac.uk/mlst/dbs/Ecoli). Sizes of the amplification products were 583, 806, 878, 932, 816, 780, and 911 bp respectively. PCR products were purified for sequencing with the QIAquick PCR purification kit (QIAGEN GmbH, Hilden, Germany). Both the forward and reverse strands were sequenced with the PCR primers set. Sequencing was performed at Macrogen, Korea (Gsan-dong Geumchen-gu, Seoul, Korea). Only MLST gene fragments with sizes around 450–530 bp were used in the eBURST and goeBURST reconstructions [2], [14]. The amplified complete nucleotide sequence for each gene was used in the phylogenetic reconstructions. The concatenated-MLST phylogenetic tree (Con-MLST) was based on a total of 5,384 bp in comparison to only 3,423 bp considered in eBURST and goeBURST. The Accession Numbers of the sequenced gene fragments are the following: KJ858688-KJ858767 (*adk*), KJ868241-KJ868320 (*fumC*), KJ868321-KJ868400 (*gyrB*), KJ868401-KJ868480 (*icd*), KJ868481-KJ868560 (*mdh*), KJ868561-KJ868640 (*purA*) and KJ868641-KJ868720 (*recA*).

### Bayesian phylogenetic analysis based on Con-MLST and detection of recombination events

Sequences of the seven genes of the MLST scheme (http://mlst.warwick.ac.uk/mlst/dbs/Ecoli) were used for the reconstruction of phylogenetic trees in our sampling. Phylogenies were obtained using a Bayesian Markov Chain Monte Carlo (MCMC) method implemented in BEAST v1.5.4 program [18]. Analysis was performed using the best-fit model of nucleotide substitution selected using the jModelTest program [19]. The TN93 model with a proportion of invariable sites plus rates vary over sites according to a gamma distribution was chosen for *icd*, *gyrB* and *recA* genes. The TN93 model with rates vary over sites according to a gamma distribution for *adk* and *mdh* genes, with a strict molecular clock model. The GTR model with a proportion of invariable sites plus rates vary over sites according to a gamma distribution for *fumC* and *purA* genes. Analysis was performed with a Bayesian Skyline piecewise-constant coalescent tree prior, using a strict molecular clock model and a random starting tree. Three separate MCMC chains were run for 400,000,000 generations, sampled every 40,000^th^ generation and combined after a 10% burn-in. BEAST output was analysed using TRACER v1.5 values of more than 200 of the effective sample size (ESS) were accepted for convergence and maximum clade credibility tree was generated after burning 10% samples with posterior probability limit >0.5 using TreeAnnotator. Species tree was established using a full sequence (5,384 bp) from the MLST genes, and species phylogroups were defined by a posterior probability >0.95 using referenced strains, known to belong to these groups.

To detect recombination events between phylogroups, the sequence of each individual gene served to construct gene trees using a Bayesian MCMC method as stated previously. Two separate MCMC chains were run for 100,000,000 generations, sampled every 10,000^th^ generation and combined after a 10% burn-in. Gene phylogroups, defined by a posterior probability >0.95, were then compared with species phylogroups. We defined a recombination event as any incongruence detected between the topology of the consensus Con- MLST tree and individual gene tree (location of individual genes in different branches with regard to the expected location).

### Determination of population structure, identification of founder clone and reconstruction of ancestral clone

The population structure of the collection of strains was obtained with the goeBURST analysis program considering the triple locus variant (TLV) approach, accessed at http://goeBURST.phyloviz.net, visualizing the relationship between sequence types (STs), previously determined by MLST using http://mlst.warwick.ac.uk/mlst/dbs/Ecoli. The founder clone, the one with most single locus variant links, was identified in ST complexes (STC) detected in the *E. coli* collection.

Evolutionary histories with inclusion of recombination events are represented in reticulate networks. Phylogenetic inferences were obtained, using the NeighborNet algorithm in SplitsTree v.4 based on spectral analysis from distances, using the same alignment both in BEAST and SplitsTree programs [20].

When the population structure could be defined using goeBURST algorithm and phylogenetic approaches, the evolutionary history of characters was reconstructed in order to infer the ancestral states using MESQUITE software V.2.75 among all STs belonging to the same STC23. Sequences from the STs phylogenetically more closely related to STC23 clade (ST1837, ST806 and ST3458) were downloaded from the MLST database and served as outgroup for the tree construction.

Ancestral reconstruction was inferred excluding STs where recombination was suspected. Intra-gene recombination was analysed using Recombination Detection Program (RDP) version 3. Later, inter-genome recombination was excluded when by comparing the topology of individual genes *versus* Con-MLST using the Tree-Puzzle program.

## Results

### Comparing multiplex PCR methods, MLST, and global optimal eBURST analysis

According to Clermont's protocol [4] of multiplex PCR for assignment of *E. coli* phylogroups, 19/80 strains in our collection were group A (23.7%), 20/80 group B1 (25%), 21/80 were group B2 (26.3%) and 20/80 group D (25%). However, following Doumith's method [5] 20/80 strains were group A (25.0%), 26/80 group B1 (32.5%), 20/80 were group B2 (25%) and 14/80 group D (17.5%) (Table S2 in File S1). Therefore, 13/80 (16.2%) strains were assigned to different phylogroups when using one or another protocol (Table 1). The most common discrepancies between both protocols were observed with the allocation of phylogroup D (6/13; 46.1%). Strains identified as phylogroup D following Clermont's protocol were assigned as either B1, A, or B2 phylogroup in Doumith's method.

**Table 1:** Phylogrouping discrepancies observed between two multiplex PCR protocols.

	Amplification pattern by Clermont's protocol				Amplification pattern by Doumith's protocol				
				Group assigned by				Group assigned by	Sequence type by
Isolate	*chuA*	*yjaA*	TSP4.C2	Clermont's protocol	*chuA*	*yjaA*	TSP4.C2	Doumith's protocol	MLST
T6	+	−	−	D	−	−	−	A	ST398
U68	−	+	−	A	−	+	+	B1	ST3372
B16	+	+	−	B2	−	+	−	A	ST540
H43	+	−	+	D	−	−	+	B1	ST359
B44	+	−	+	D	−	−	+	B1	ST602
E9	+	+	+	B2	−	+	+	B1	ST2973
C17	+	−	+	D	−	−	+	B1	ST58
T22	+	−	+	D	−	−	+	B1	ST58
T30	+	+	+	B2	−	+	+	B1	ST155
E48	+	+	+	B2	−	+	+	B1	ST345
E76	+	−	+	D	+	+	+	B2	ST537
E42	−	−	+	B1	+	+	+	B2	ST978
E33	−	−	+	B1	+	+	+	B2	ST3366

**Figure 1:**
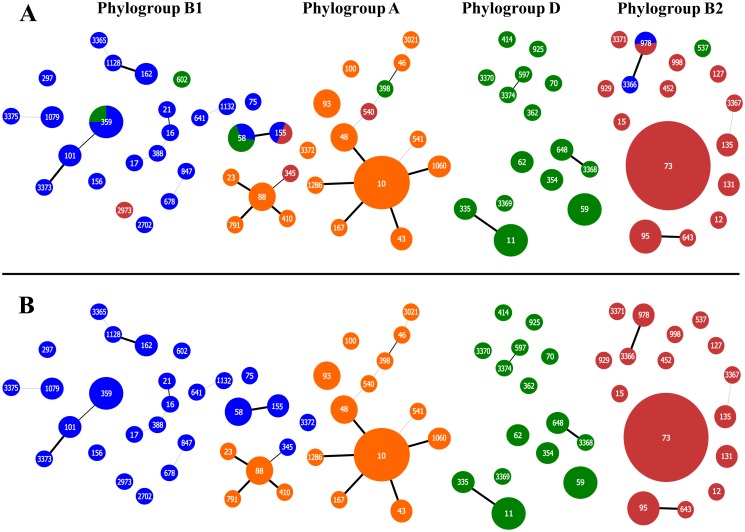
*E. coli* population structure visualized using goeBURST. A) Phylogroups identified based on Clermont's protocol. B) Phylogroups identified based on Doumith's protocol. The phylogroups are identified by colors: blue, orange, green and red correspond to B1, A, D and B2 respectively. ST numbers assigned are overprinted. STs with ≤3 differences (TLV) are connected by lines of different thickness (thicker line corresponds to single locus variant, SLV).

Following the recommendations of MLST scheme, 50 different STs were identified in our collection; the inferred population structure was visualized using goeBURST where the phylogroups identified based on Clermont and Doumith's schemes are overprinted (Fig. 1). According to the obtained representation, Doumith's scheme offers more coherent assignment that the previous Clermont's protocol. Close to 83% of the STs (10/12) in which phylogroup discrepancies were found between both schemes were apparently better assigned using Doumith's recommendations according to population structure inferred by goeBURST representation. In any case it is patent that this representation offers a quite dispersed population structure inside each lineage with very few well-defined relations among different STs, and is obviously insufficient to detect evolutionary relationships among them. For instance, only two primary founder STs (in STC23 and in STC10) could be suspected among the strains belonging to phylogroup A (Fig. 1).

### *E. coli* lineages and evolutionary relationships obtained by phylogenetic reconstruction analysis *versus* multiplex PCR and goeBURST protocols

**Figure 2:**
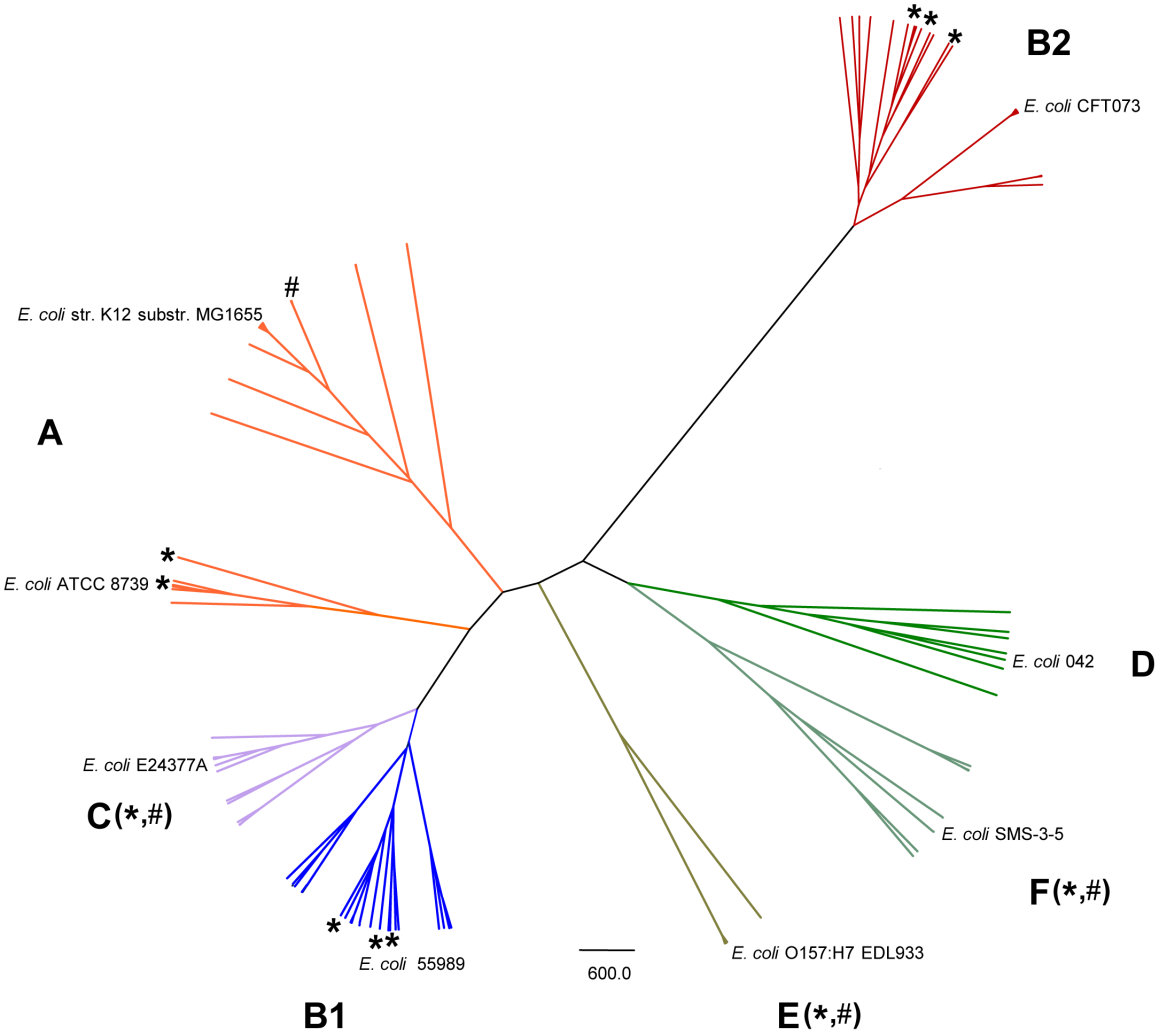
Phylogenetic tree based on concatenated MLST-genes (Con-MLST) using BEAST v1.5.4 program. Phylogroups were established with posterior probability >0.95. Discrepancies between multiplex PCR and phylogeny are shown as *(discrepancies using Clermont's protocol) and # (discrepancies using Doumith's protocol). The discrepancies affecting members belonging to non-detected phylogroups (C, F and E) using multiplex PCRs are shown close to the character defining the phylogroup. Forty-eight sequences of reference strains downloaded from GenBank were used in the analysis, but one strain for each phylogroup is shown.

The phylogenetic tree of seven concatenated MLST-genes (5,384 bp) was used to classify our *E. coli* collection of strains. The Con-MLST-genes phylogenetic reconstruction fully depicted the seven macro-evolutionary lineages of *E. coli sensu stricto* (A, B1, C, B2, D, E and F) [7]–[12], allowing a more accurate definition of lineages (Fig. 2) in comparison with the pattern obtained with the concatenated fragment of 3,423 bp used in eBURST (Fig. S1). It now became patent that *E. coli* strains of this collection were distributed in seven evolutionary lineages, and strains belonging to phylogroups C (14 strains), E (1 strain) and F (8 strains) were identified (Fig. 2). According to the new reclassification observed with Con-MLST phylogenetic reconstruction, globally Clermont's protocol reached 38.7% of misclassified phylogroups in our collection; whereas following Doumith's protocol the discrepancies were 30%. These misclassifications are so high because these multiplex PCR protocols were designed to classify only the main phylogroups (A, B1, B2 and D). According to the Bayesian tree, the strains allocated in phylogroup C were previously identified as A (6/14) and B1 (8/14) using Doumith's scheme, or as A (6/14), B1 (4/14), B2 (2/14) or D (2/14) applying Clermont's protocol. Moreover the strains assigned to E and F phylogroups corresponded to those identified as phylogroup D using any multiplex-PCR protocol. When the results were re-analysed excluding non-detected minority phylogroups, the misclassifications were 14.0% and 1.7% for Clermont and Doumith protocols respectively, confirming more accurate allocation following Doumith's protocols as was suggested in a previous section (Fig. 2).

**Figure 3:**
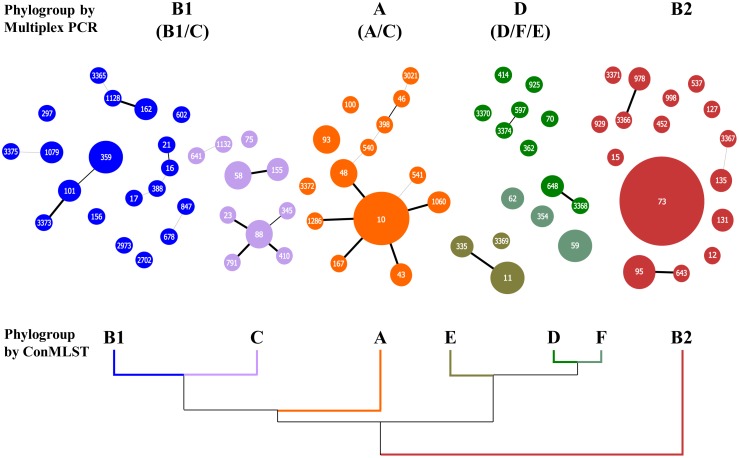
*E. coli* population structure of our collection visualized by using goeBURST in combination with Con-MLST phylogenetic analysis. Up: Four phylogroups identified based on multiplex PCR techniques. Down: Seven phylogroups based on Con-MLST phylogenetic analysis.

When considering the results obtained with phylogenetic reconstruction, the population structure represented in Figure 1 could be re-interpreted with more precision, combining an approach between phylogeny and goeBURST (Fig. 3).

**Figure 4:**
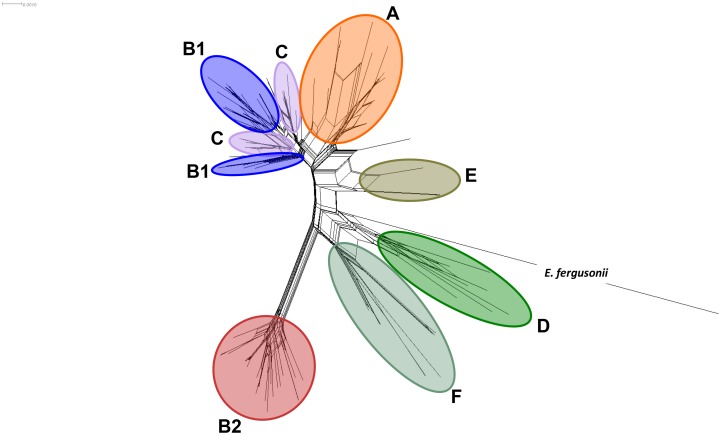
Network phylogenetic analysis based on Con-MLST obtained with NeighborNet algorithm in SplitsTree v.4. This representation allows inferring more complex interactions among the strains than goeBURST. The main phylogroups are differentiated in coloured circles. Members belonging to phylogroup C are located in two positions in the tree as two different patterns of recombination between B1 and A phylogroups were observed (see Figure 5).

In order to improve our understanding of the evolutionary relationships among strains of this collection a new approach was generated using NeighbourNet algorithm (Fig. 4). This figure shows a high rate of interaction at different levels in and between different lineages, which enables a better establishment of relationships among all sequences than with goeBURST [3]. Reticulations in the diagram are suggestive of recombination events (although other options such as convergent evolution or lack of resolution can be suspected). While strains assigned to phylogroup B2 mostly indicated only intraclade recombination, strains belonging to phylogroups A, B1 and C showed the highest rate of homoplasy. These results emphasize the important weight of recombination in shaping the evolutionary relationships between *E. coli* isolates.

### Discrepancies between phylogenetic and non-phylogenetic analysis of *E. coli* population structure are due to recombination events

**Figure 5:**
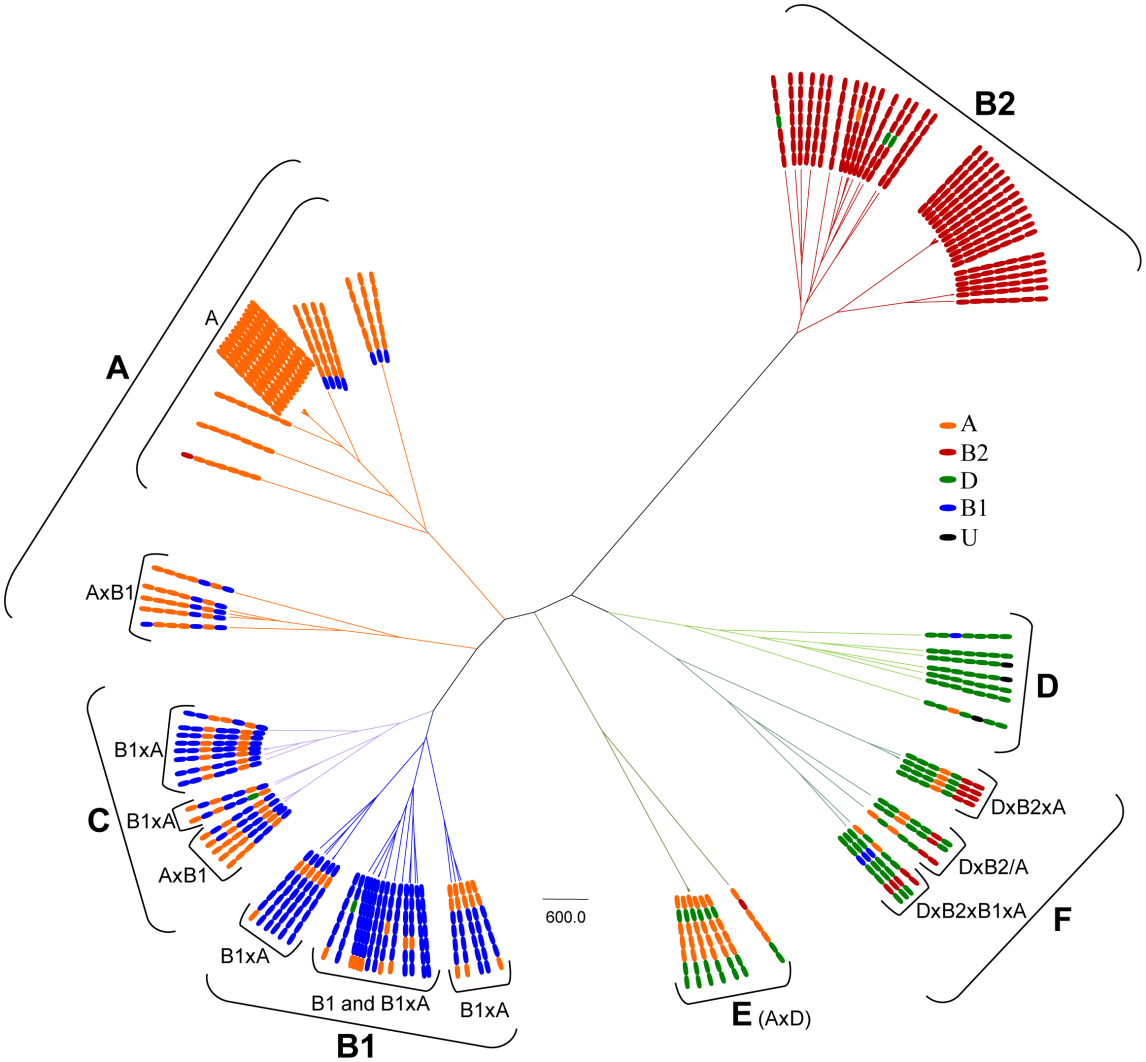
Consensus tree based on Con-MLST; overprinted, the concatenated trees of individual genes used in MLST analysis. Maximum clade credibility tree was generated after burning 10% samples with posterior probability limit >0.5 using TreeAnnotator. Each segment in the figure corresponds to the seven genes used in MLST scheme in the following order: *adk-icd-fumC-recA-mdh-gyrB-purA*, being *adk* gene the inner segment. Recombination events in the different individual genes are shown. Phylogroups (A, B1, D, B2; U = unknown) were established with posterior probability >0.95. This approximation infers that most of the novel lineages could be the result of recombination events.

Phylogenetic trees obtained using individual genes from MLST schema were compared with the consensus Con-MLST tree revealing phylogenetic incongruences. We interpreted these incongruences as resulting from acquisition of exogenous DNA (Fig. 5). Table 2 shows the percentage of incongruences for lineages and also the impact of recombination in individual MLST genes. The B2 phylogroup showed the lowest intergroup recombination frequencies (1.6%), while B1 phylogroup had the highest ones (17.7%) among non-recombinant lineages (A, B1, B2 and D). On the other hand, the very high frequency of incongruences for several genes in minority phylogroups (C, E and F) suggested that these phylogroups could be the result of ancient recombination events. According to these data, the phylogroup C could be a new lineage derived from recombination events that have occurred between members of phylogroups B1 and A. The results explain why phylogroup C was misclassified as phylogroup A (8 strains) or B1 (6 strains) using the multiplex PCR approach (see previous section). Similarly, we were able to identify strains belonging to phylogroup F showing promiscuous gene interactions with phylogroup D and other phylogroups. The high frequency of incongruences observed within phylogroup F identifies at least two different ancestral recombination events in this phylogroup, suggesting the possibility of emergence of a new branch (Fig. 5). To avoid overestimation in the recombination frequencies in C, E and F phylogroups, we only recorded as true recombination the cases where the donor phylogroup was different to the phylogroup involved in ancestral recombination (Table 2).

**Table 2:** MLST genes recombination frequencies inferred in the different *E. coli* phylogenetic groups.

	Phylogenetic group	*adk*	*icd*	*fumC*	*recA*	*mdh*	*gyrB*	*purA*	Total
Class of phylogenetic group	(number of strains)	%	%	%	%	%	%	%	%
Main groups (Non-Recombinant)	B2 (n = 35)	0	0	0	8.6	2,9	0	0	1.6
	B1 (n = 25)	20	44	4	4	8	0	44	17.7
	A (n = 28)	42.9	0	17.9	0	0	0	7.1	9.7
	D (n = 7)	0	0	28.6	0	14.3	0	28.6	10.2
	TOTAL (n = 95)	17.9	11.6	8.4	4.2	4.2	0	15.8	8.9
Minority groups (Recombinant)	C (n = 15)	6.7	6.7^b^	0	6.7	0^b^	20	46.7^c^	12.4
	F (n = 11)	18.2	0	63.6	36.4	0	27.3^b^	54.5^d^	28.6
	E (n = 7)	0	14.3^b^	0	0	0	0^b^	0^b^	2.1
	TOTAL (n = 33)	9.1	6.1	21.2	15.1	0	18.2	39.4	16.4

Recombination frequency was inferred as n° of phylogenetic incongruences with respect to total number of strains in each phylogroup. ^a^ *purA* gene belongs to phylogroup A in seven strains and in eight of them belongs to phylogroup B1. ^b^ *purA* gen belongs to phylogroup D in five strains and in six of them belongs to phylogroup B2.

Differences were observed even among non-recombinant lineages in the recombination frequencies for the different MLST genes. *gyrB, mdh* and *recA* genes showed the lowest frequencies of incongruences (0%, 4.2% and 4.2% respectively), whereas *adk* and *purA* genes showed the highest ones (17.9% and 15.8% respectively). These results revealed the high interchange frequency affecting these genes in both recombinant and non-recombinant lineages, suggesting that the selection of *purA* and *adk* genes should be re-evaluated for the purposes of future MLST-typing.

### Tracing the hypothetical evolutionary trajectories of the strains using eBURST and phylogenetic reconstructions

**Figure 6:**
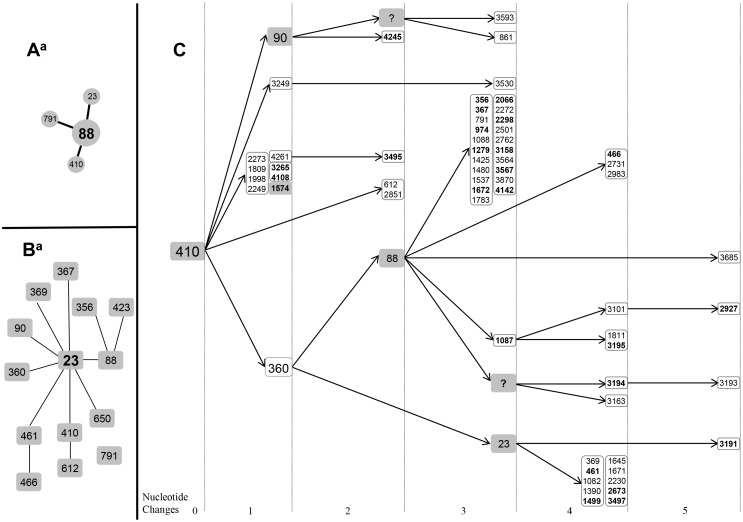
Hypothetical evolutionary reconstruction of founder clone in STC23. A) eBURST of members belonging to STC23 present in our collection. B) eBURST of complete clonal complex STC23 according to information available in MLST database. C) Phylogenetic reconstruction of ancestral state in STC23 using MESQUITE v. 2.75. Numbers in bold type indicate non-synonymous changes. STs in grey box correspond to founder. According to this analysis ST410 could be the ancestral clone and ST88 a sub-founder clone derived from ST360, which was not recognised in eBURST as founder clone, ^a^eBURST shows the relation between ST with only one allelic variation (SLV). Box with? symbol indicates an unknown hypothetical ancestral.

In the combined representation using phylogeny and goeBURST, only A and C phylogroups showed a well-defined population structure, thus allowing identification of the founder clones (Fig. 3). According to the simplest evolutionary model, the central position in the population structure corresponds to the founder clone. In phylogroup C, the ST88 could be considered the founder clone of ST23, ST791, ST410 (Fig. 6A), but at the MLST database the founder clone corresponds to ST23 belonging to STC23 (Fig. 6B). This discrepancy could be the consequence of the low representation of STC23 in our collection. In order to resolve this discrepancy, all STs from the MLST database were reanalyzed (Fig. S2 and Fig. S3), using MESQUITE (see material and methods) for ancestor reconstruction, but surprisingly a new ancestor clone was found (Fig. 6C). The ancestral position was now occupied by ST410. In fact ST23 was identified as a secondary founder clone of ST360 (evolved variant of ST410). Now ST360 was ancestral clone of both secondary founders ST23 and ST88 both of which had different evolutionary trajectories.

## Discussion

*Escherichia coli* is a good example of host-adaptable bacterial species depicting a wide eco-pathological diversity. The population structure of *E. coli* certainly reflects the ecogenetics of this organism, which is highly dependent on recombinational and mutational events, not only involving adaptive mobile genes, but probably also housekeeping genes [1], [7], [8], [21]. Misclassifications in *E. coli* lineages or phylogenetic groups could be not only the consequence of non-specific amplification (point mutations) but also of horizontal gene transfer. In this paper a comparative analysis of two different techniques (multiplex PCR plus MLST and Con- and individual-MLST-phylogeny) for phylogroup assignment and population structure analysis was performed on a selected *E. coli* strain collection.

In order to determine the impact of the mutations we compared Doumith's multiplex PCR protocol [5] updating the design of the primers used in the 2000 Clermont's method [4], reducing unspecific annealing and promoting specific amplification, thus improving coverage. Non-specific amplifications (as for an acetyl-hydrolase gene) using conventional Clermont's protocol have been reported for B1 isolates [22], but this protocol has also occasionally failed in the amplification of *chu*A and/or *yja*A [5], [23], [24], [25], [26]. In fact, in our series Clermont's multiplex PCR approach provides a worse assignment (14.0% misclassified) than Doumith's schema (1.7%), when C, E and F lineages were excluded. Both protocols exhibited the highest discrepancies in the assignment of the phylogroup D, as was previously observed [5], [6]. Of course a limitation of the tested multiplex PCR techniques is that they cannot detect the minority phylogroups (C, E, F) and *Escherichia* clade I, and the correct identification of minority or recombinant phylogroups is clinically relevant [27]. There was a high rate of wrong classifications in our collection, both for the tested Clermont's (38.7%) and Doumith's protocols (30%), higher than in previous publications [5], [6]. Therefore, if in the sample under study there is a substantial proportion of the minority phylogroups the reliability for assigning relations among the members of the population structure could be significantly impaired [28]. Does Con-MLST phylogeny reflect whole genome phylogeny? Although whole genome analysis should give the most precise phylogenetic reconstruction, it is currently still out of reach for routine molecular epidemiology. Though any gene could be involved in horizontal gene transfer events [29], recombination has not occurred at a sufficient level to disrupt the phylogenetic signal present in whole genome datasets [17]. Phylogenetic analysis based on Con-MLST showed better resolution than concatenated fragment used in eBURST respect to whole genome analysis (Fig. 2 and Fig. S1). Likewise it is remarkable how strains belonging to clonal complexes (as STC66 and STC23) clearly assigned to phylogroup C by phylogenetic reconstruction were identified as members of phylogroup A by multiplex PCR in previous works [10], [27], assigned as B1 in MLST web page, and as AxB1 in other publications [30]. This illustrates the difficulty in the allocation of strains with recombinant phylogenetic origin.

The population structure *E. coli* using goeBURST based on MLST sequences offers advantages in the characterization of acute outbreaks, however a limitation of this approach is observed in non-epidemic situations, in which population structure is very dispersed and there is only scarce relation between STs. Recombination events play an important role in the evolution of *E. coli* [26], [31], [32], [33], for instance individual genes-MLST phylogeny confirmed a high recombination frequency among *E. coli* strains included in our collection. The most frequent gene exchange was observed between the seemingly evolutionary close phylogroups A and B1, in which the generation and selection of new recombinant forms is more probable, such as C or F phylogroups [30]. Therefore NeighbourNet phylogeny helps us to understand the complex population structure of *E. coli* (Fig. 4), because the reticulations could suggest the presence of recombination events.

Interestingly, the recombination events do not have a similar distribution in the different phylogroups. Members of phylogroup B2 showed 10-fold lower recombination events than phylogroup B1. These results agreed with recently published whole genome analysis [25], [34]. Figure 2 shows that branches corresponding to phylogroup B2 and E are very isolated with respect to other *E. coli* lineages, illustrating few opportunities of recombination. In fact strains of phylogroup B2 tend not to coexist with strains of other phylogroups [35]. Genetic isolation could explain the excellent allocation of strains belonging to phylogroup B2 when using multiplex PCR protocols (85% for Clermont's method and 100% for Doumith's method, respectively). Those collections containing a high proportion of more recombinogenic strains will be less precisely analyzed by multiplex PCR [28].

Although *a priori* the main limitations of multiplex PCR+ eBURST approaches are the non-identification of recombinant lineages and very dispersed population structure, the eBURST analysis can eventually generate discrepancies in the identification of ST ancestor clones and in the recognition of evolutionary trajectories, with consequences in the characterization of local clonal invasions and outbreaks. For instance, among the STs belonging to phylogroup C, two different founder clones were identified according to MLST database and our collection (ST23 and ST88 respectively). In order to resolve this discrepancy, all STs from the MLST database were reanalyzed (Fig. S2 and Fig. S3). Following the eBURST algorithm, ST88 was identified as founder clone (47 SLV); however, the ancestral phylogenetic reconstruction identified ST410 as ancestral clone. Thus, the evolutionary trajectories showed that ST88 and ST23 were clones evolved from ST360 which derived directly from ST410 (Fig. 6C). Our study suggests that ancestor's reconstruction might be relevant in the characterization of the emergent local clonal invasions and epidemic outbreaks. In this respect, Con-MLST phylogenetic analysis could help eBURST analysis to characterize the evolutionary dynamics in clonal invasions, expansions and outbreaks, especially those caused by pathogenic strains that can be submitted to a fast evolution. Recombination constitutes a severe limitation for the construction of reliable phylogenetic trees. In fact, previously to perform a phylogenetic tree, recombination must be excluded to the best of our possibilities.

The first caveat of our study is the relatively low sample size, but our collection has a well-balanced representation of all phylogroups. A second limitation in our comparative study was the lack of inclusion of the recently described multiplex PCR method developed by Clermont *et al*. which is able to correctly assign almost all lineages including non-recombinant phylogroups [11]. Nevertheless, even in the best possible scenario, multiplex PCR techniques are expected to fail in allocating recombinant variants, which might give rise to successful combinations between very evolutionary related phylogroups such as A and B1.

Finally, we suggest a practical analytical approach based in three successive complexity steps. First, assignment of phylogroups following the Doumith's protocol discriminating among A/B1/C, D/F/E and B2 lineages. Second, sequencing of MLST genes helping to identify specific successful clones in outbreaks or clonal expansions (goeBURST). Third, using phylogenetic reconstructions for the precise assignment of lineages, the characterization of population networks and evolutionary dynamics, especially if dealing with populations involving a high proportion of recombinants, fast-evolving strains, or isolates obtained along extended periods of time. In spite of a slightly higher complexity, we recommend the application of phylogenetic approaches based on Con-MLST, as an affordable alternative to the more expensive high-resolution studies based on complete genome sequencing.

## Supporting Information

Figure S1 **Phylogenetic tree based on concatenated fragment of 3,423 bp used in eBURST.** Phylogroups were established with posterior probability >0.95. Forty-eight sequences of reference strains downloaded from GenBank were used in the analysis, but one strain for each phylogroup is shown. The phylogenetic tree was obtained using BEAST v1.5.4 program.
(TIF)

Figure S2 **eBURST obtained with all STs available in MLST database closely related to STC23.** In accordance with the eBURST roles, ST88 is presumed to be the founder clone (maximum number of SLVs) with 47 SLV. However the number of SLV only reveals the clone with the most diversification rate, but not necessarily the ancestor clone.
(TIF)

Figure S3 **Bayesian phylogenetic tree with all STs available in MLST database closely related to STC23.** The known STs most phylogenetically related to STC23 clade was established as an outgroup of the tree, which was necessary in the ancestral reconstruction approach. The STs into boxes correspond to defined ST in MLST webpage as clonal complex STC23. Maximum clade credibility tree was generated after burning 10% samples with posterior probability limit >0.5 using TreeAnnotator.
(TIF)

File S1 **Supporting tables.**
(DOC)
