# Correction: Recombination Blurs Phylogenetic Groups Routine Assignment in *Escherichia coli*: Setting the Record Straight

**DOI:** 10.1371/journal.pone.0113532

**Published:** 2014-11-10

**Authors:** The PLOS ONE Staff

There are errors in the funding statement. Please see the correct funding statement below.

MCT and FB are supported by the European Union (EvoTAR-FP7-Health-2011-282004), JCG by a grant of the Ministerio de Economía y Competitividad, Instituto de Salud Carlos III, Spain, (PI12/00567), and European Union (EvoTAR-FP7-Health-2011-282004). JMGA is supported by a fellowship from the Regional Government of Madrid in Spain (PROMT-S2010/BMD2414).The funders had no role in study design, data collection and analysis, decision to publish, or preparation of the manuscript.
