# Supplementary figures and images for: Recombination Blurs Phylogenetic Groups Routine Assignment in Escherichia coli: Setting the Record Straight
Source: PLoS One. 2014 Aug 19;9(8):e105395. doi: 10.1371/journal.pone.0105395 (PMC4138120; doi:10.1371/journal.pone.0105395)

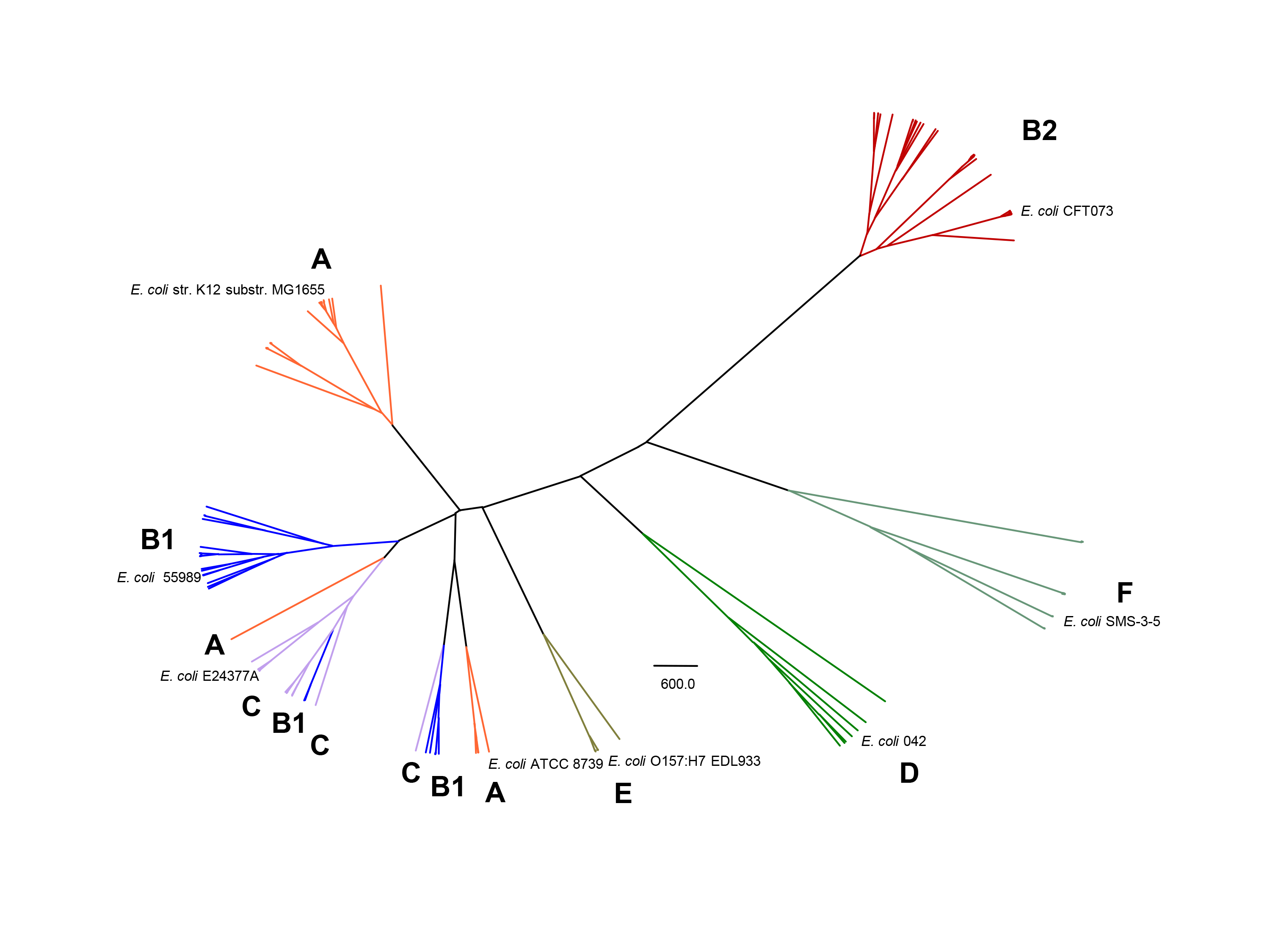

Supplement: Figure S1 — Phylogenetic tree based on concatenated fragment of 3,423 bp used in eBURST. Phylogroups were established with posterior probability >0.95. Forty-eight sequences of reference strains downloaded from GenBank were used in the analysis, but one strain for each phylogroup is shown. The phylogenetic tree was obtained using BEAST v1.5.4 program. (TIF) [file pone.0105395.s001.tif]

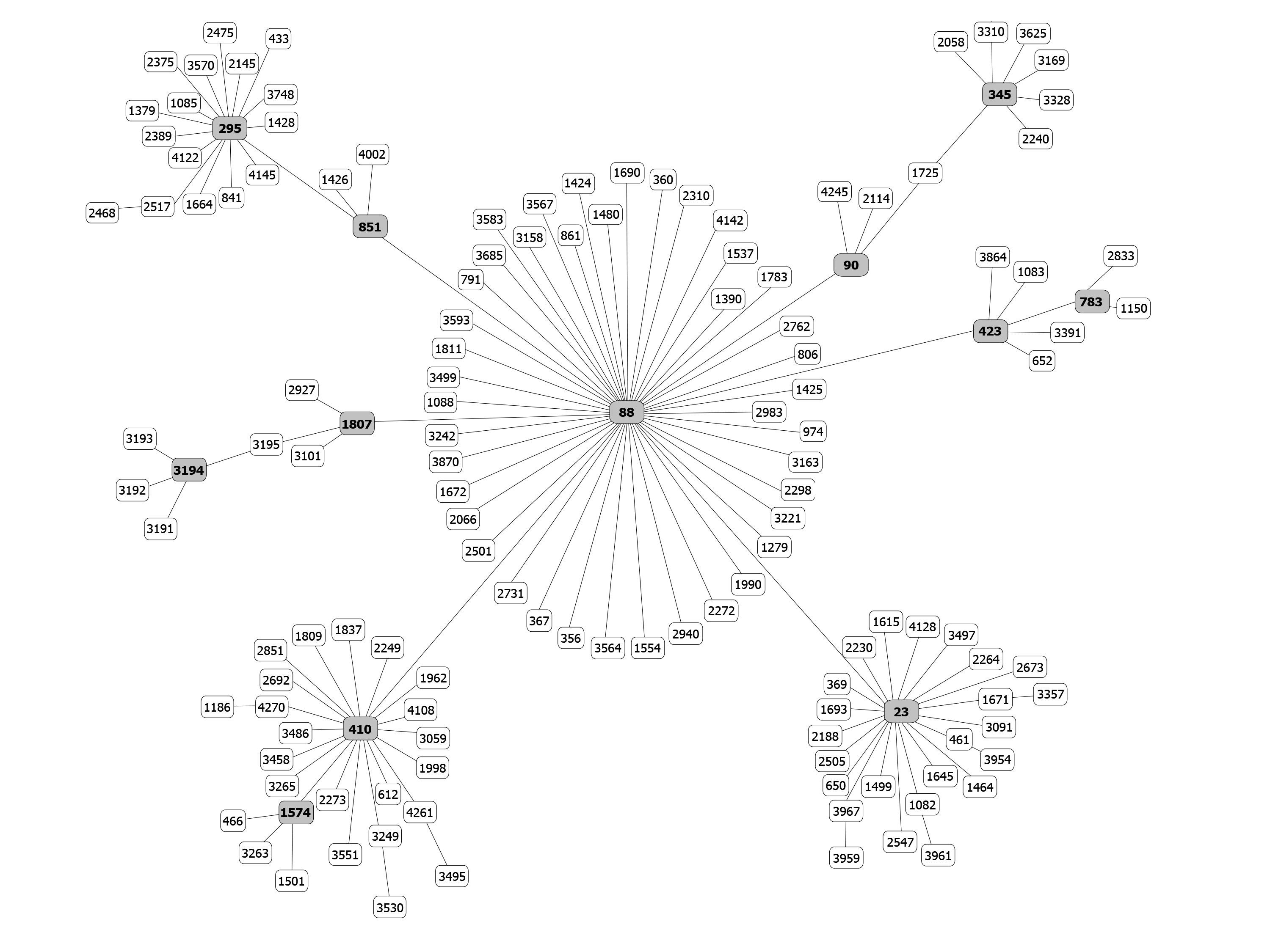

Supplement: Figure S2 — eBURST obtained with all STs available in MLST database closely related to STC23. In accordance with the eBURST roles, ST88 is presumed to be the founder clone (maximum number of SLVs) with 47 SLV. However the number of SLV only reveals the clone with the most diversification rate, but not necessarily the ancestor clone. (TIF) [file pone.0105395.s002.tif]

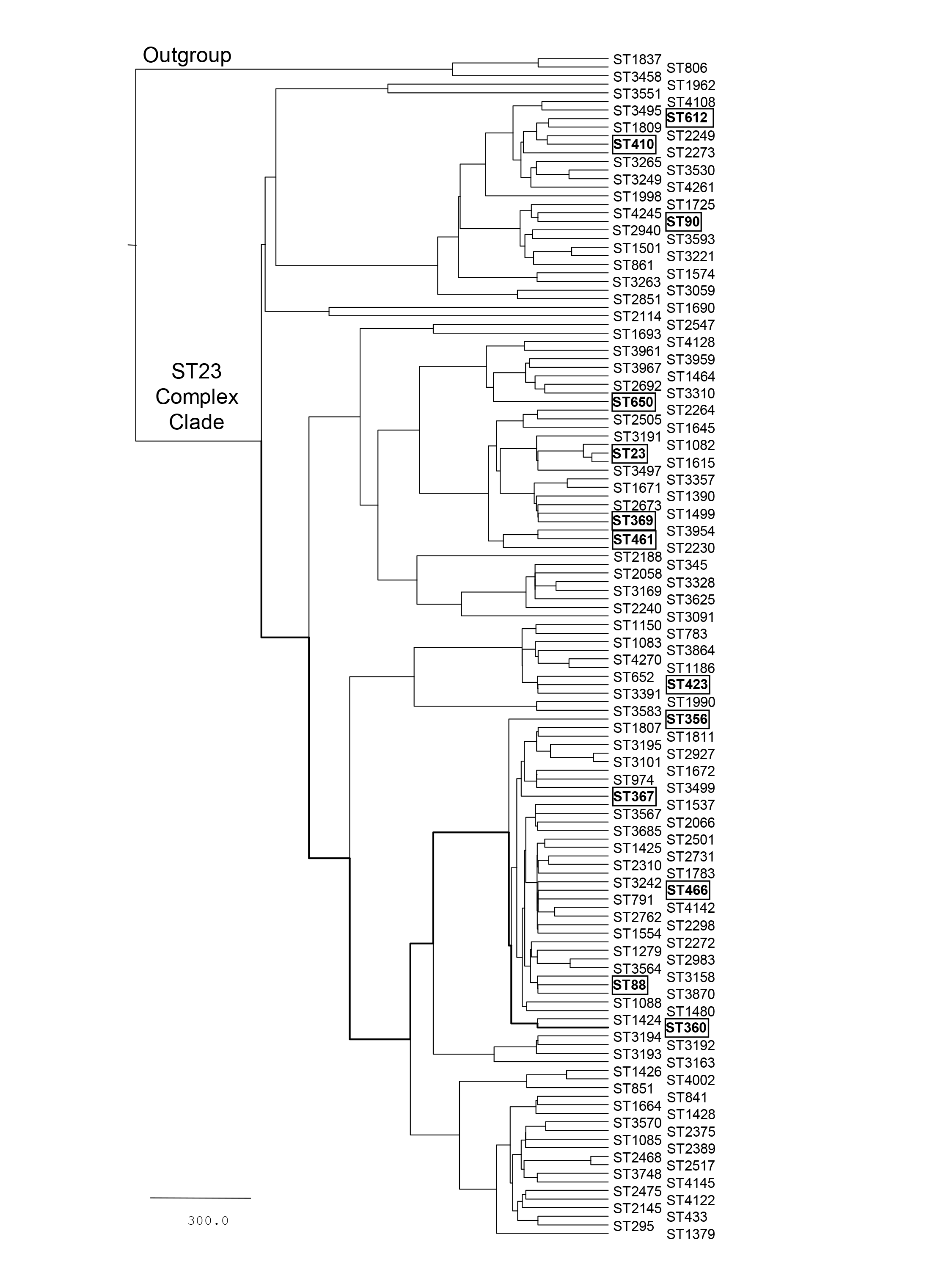

Supplement: Figure S3 — Bayesian phylogenetic tree with all STs available in MLST database closely related to STC23. The known STs most phylogenetically related to STC23 clade was established as an outgroup of the tree, which was necessary in the ancestral reconstruction approach. The STs into boxes correspond to defined ST in MLST webpage as clonal complex STC23. Maximum clade credibility tree was generated after burning 10% samples with posterior probability limit >0.5 using TreeAnnotator. (TIF) [file pone.0105395.s003.tif]
