# Supplementary material for: Recombination Blurs Phylogenetic Groups Routine Assignment in Escherichia coli: Setting the Record Straight
Source: PLoS One. 2014 Aug 19;9(8):e105395. doi: 10.1371/journal.pone.0105395 (PMC4138120; doi:10.1371/journal.pone.0105395)
Supplement: File S1 — Supporting tables. (DOC) [file pone.0105395.s004.doc]

**Supporting Information**

**Recombination Blurs Phylogenetic Group Routine Assignment in *Escherichia coli*: Setting the Record Straight**

Turrientes MC, González-Alba JM, del Campo R, Baquero MR, Cantón R, Baquero F, Galán JC

**Table S1**. **The forty-eight *Escherichia coli* fully sequenced strains included in this study**. ^a^HC, Human commensal, AIEC, adherent invasive *E. coli*; APEC, avian *E. coli*; EAEC, enteroaggregative *E. coli*; EHEC, enterohemorrhagic *E. coli*, EPEC, enteropathogenic *E coli*; ETEC, enterotoxigenic *E. coli*; ExPEC, extraintestinal pathogenic *E. coli,* UPEC, uropathogenic *E. coli*. ^b^Extracellular pathogenic *E. coli*. ^c^NCBI, National Center for Biotechnology Information

|  |  |  | **Accesion** | **Phylogenetic** |
| --- | --- | --- | --- | --- |
|  |  |  | **Number.** | **group by** |
| **Strain** | **Pathotype^a^** | **Clinical condition^a^** | **(INSDC)^c^** | **Con-MLST** |
| *E. coli* str. K12 substr. MG1655 | K12 | Commensal | U00096.2 | A |
| *E. coli* HS | HC | Commensal | CP000802.1 | A |
| *E. coli* ATCC 8739 | K12 | Commensal | CP000946.1 | A |
| *E. coli* BL21 (DE3) | B | Commensal | CP001509.3 | A |
| *E. coli* B str. REL606 | B | Commensal | CP000819.1 | A |
| *E. coli* BL 21 Gold | B | Commensal | CP001665.1 | A |
| *E. coli* UMNK88 | K88 | ETEC (pig) | CP002729.1 | A |
| *E. coli* DH1 | K12 | Commensal | CP001637.1 | A |
| *E. coli* K12 str. DH10B | K12 | Commensal | NC_010473.1 | A |
| *E. coli* K12 str. W3110 | K12 | Commensal | AP009048.1 | A |
| *E. coli* BW2952 | K12 | Commensal | CP001396.1 | A |
| *E. coli* P12b | K12 | Commensal | CP002291.1 | A |
| *E. coli* ETEC H10407 | ETEC | ETEC | FN649414.1 | A |
| *E. coli* O26:H11 str. 11368 | O26 EHEC | EHEC | AP010953.1 | B1 |
| *E. coli* SE11 | HC | Commensal | AP009240.1 | B1 |
| E. coli O111:H str. 11128 | O111 EHEC | EHEC | AP010960.1 | B1 |
| *E. coli* KO11FL | W | Laboratory strain. | CP002516.1 | B1 |
| *E. coli* W ATCC 9637 | W | Environmental (soil) | CP002185.1 | B1 |
| *E.coli* O103:H2 str. 12009 | O103 EHEC | EHEC | AP010958.1 | B1 |
| *E. coli* IAI1 | O:8 | Commensal | CU928160.2 | B1 |
| *E. coli* 55989 | EAEC | EAEC | CU928145.2 | B1 |
| *E. coli* E24377A | ETEC | ETEC | CP000800.1 | C |
| *E. coli* O55:H7 str. RM12579 | O55 EHEC | EHEC | CP003109.1 | E |
| *E. coli* O55:H7 str.CB9615 | O55 EHEC | EPEC (diarrhoea) | CP001846.1 | E |
| *E. coli* O157:H7 str.Sakai | O157 EHEC | EHEC | BA000007.2 | E |
| *E. coli* O157:H7 str. EC4115 | O157 EHEC | EHEC | CP001164.1 | E |
| *E. coli* O157:H7 str. TW14359 | O157 EHEC | EHEC | CP001368.1 | E |
| *E. coli* O157:H7 EDL933 | O157 EHEC | EHEC (food; ground beef) | AE005174.2 | E |
| *E. coli* 042 | EAEC | EAEC | FN554766.1 | D |
| *E. coli* UMN026 | O:7 ExPEC | ExPEC (UPEC/cystitis)^b^ | CU928163.2 | D |
| *E. coli* SMS-3-5 | Multidrug resistant | Environmental | CP000970.1 | F |
| *E. coli* O7:K1 str. CE10 | K1 ExPEC | ExPEC (Meningitis) | CP003034.1 | F |
| *E. coli* IAI39 | K1 ExPEC | ExPEC (UPEC/pyelonefritis)^b^ | CU928164.2 | F |
| EcoliUM146 | AIEC | AIEC | CP002167.1 | B2 |
| *E. coli* APEC O1 | APEC | APEC (ExPEC/avian) | CP000468.1 | B2 |
| *E. coli* S88 | O45 ExPEC | ExPEC (Meningitis )^b^ | CU928161.2 | B2 |
| *E. coli* IHE3034 | ST95 ExPEC | ExPEC (Meningitis ) | CP001969.1 | B2 |
| *E. coli* ABU 83972 | Asymptomatic | ExPEC (bacteriuria) | CP001671.1 | B2 |
| *E. coli* CFT073 | ExPEC | ExPEC (UPEC/ pyelonephritis) | AE014075.1 | B2 |
| *E. coli* Di14 | ExPEC | ExPEC (UPEC) | CP002212.1 | B2 |
| *E. coli* Di2 | ExPEC | ExPEC (UPEC) | CP002211.1 | B2 |
| *E. coli* O127:H6 str. E2348/69 | O127 EPEC | EPEC | FM180568.1 | B2 |
| *E. coli* SE15 | HC | HC | AP009378.1 | B2 |
| *E. coli* NA114 | ExPEC | ExPEC (UPEC) | CP002797.2 | B2 |
| *E. coli* LF82 | AIEC | AIEC | CU651637.1 | B2 |
| *E. coli* O83:H1 NRG857c | AIEC | AIEC | CP001855.1 | B2 |
| *E. coli* ED1a | O81 | Commensal | CU928162.2 | B2 |
| *E. coli* 536 | O6 ExPEC | ExPEC (UPEC/pyelonephritis ) | CP000247.1 | B2 |

**Table S2**. A list of STs of eighty strains involved in the study and the assignation of phylogenetic groups according to Clermont or Doumith schema based on multiplex PCR results.

|  | **Amplification pattern by** | | |  | **Amplification pattern by** | | |  |  |
| --- | --- | --- | --- | --- | --- | --- | --- | --- | --- |
|  | **Clermont`s protocol** | | | **Group assigned by** | **Doumith`s protocol** | | | **Group assigned by** | **Sequence type by** |
| **Isolate** | *chuA* | *yjaA* | TSP4.C2 | **Clermont’s protocol** | *chuA* | *yjaA* | TSP4.C2 | **Doumith’s protocol** | **MLST** |
| RYC-H22 | - | + | - | A | - | + | - | A | ST10 |
| RYC-U11 | - | + | - | A | - | + | - | A | ST167 |
| RYC-T10 | - | + | - | A | - | + | - | A | ST43 |
| RYC-C24 | - | + | - | A | - | + | - | A | ST1286 |
| RYC-T12 | - | + | - | A | - | + | - | A | ST10 |
| RYC-C74 | - | + | - | A | - | + | - | A | ST10 |
| RYC-T7 | - | + | - | A | - | + | - | A | ST43 |
| RYC-E5 | - | + | - | A | - | + | - | A | ST10 |
| RYC-B13 | - | + | - | A | - | + | - | A | ST541 |
| RYC-T6 | + | - | - | D | - | - | - | A | ST398 |
| RYC-U68 | - | + | - | A | - | + | + | B1 | ST3372 |
| RYC-U14 | - | + | - | A | - | + | - | A | ST48 |
| RYC-S22 | - | + | - | A | - | + | - | A | ST48 |
| RYC-S30 | - | + | - | A | - | + | - | A | ST48 |
| RYC-B16 | + | + | - | B2 | - | + | - | A | ST540 |
| RYC-T60 | - | - | + | B1 | - | - | + | B1 | ST2702 |
| RYC-CV18 | - | - | + | B1 | - | - | + | B1 | ST162 |
| RYC-E41 | - | - | + | B1 | - | - | + | B1 | ST162 |
| RYC-CV77 | - | - | + | B1 | - | - | + | B1 | ST3375 |
| RYC-S12 | - | - | + | B1 | - | - | + | B1 | ST3365 |
| RYC-U8 | - | - | + | B1 | - | - | + | B1 | ST101 |
| RYC-CV590 | - | - | + | B1 | - | - | + | B1 | ST359 |
| RYC-CV29 | - | - | + | B1 | - | - | + | B1 | ST359 |
| RYC-CV61 | - | - | + | B1 | - | - | + | B1 | ST359 |
| RYC-H43 | + | - | + | D | - | - | + | B1 | ST359 |
| RYC-S2 | - | - | + | B1 | - | - | + | B1 | ST101 |
| RYC-B44 | + | - | + | D | - | - | + | B1 | ST602 |
| RYC-B23 | - | - | + | B1 | - | - | + | B1 | ST3373 |
| RYC-E1 | - | - | + | B1 | - | - | + | B1 | ST847 |
| RYC-E9 | + | + | + | B2 | - | + | + | B1 | ST2973 |
| RYC-E39 | - | - | + | B1 | - | - | + | B1 | ST297 |
| RYC-S1 | - | - | + | B1 | - | - | + | B1 | ST388 |
| RYC-CV920 | - | - | + | B1 | - | - | + | B1 | 641 |
| RYC-CV80 | - | - | + | B1 | - | - | + | B1 | ST58 |
| RYC-C17 | + | - | + | D | - | - | + | B1 | ST58 |
| RYC-T22 | + | - | + | D | - | - | + | B1 | ST58 |
| RYC-T30 | + | + | + | B2 | - | + | + | B1 | ST155 |
| RYC-T35 | - | - | + | B1 | - | - | + | B1 | ST155 |
| RYC-DA | - | + | - | A | - | + | - | A | ST410 |
| RYC-U15 | - | + | - | A | - | + | - | A | ST88 |
| RYC-C49 | - | + | - | A | - | + | - | A | ST88 |
| RYC-U5 | - | + | - | A | - | + | - | A | ST23 |
| RYC-E3 | - | + | - | A | - | + | - | A | ST88 |
| RYC-E34 | - | + | - | A | - | + | - | A | ST791 |
| RYC-E48 | + | + | + | B2 | - | + | + | B1 | ST345 |
| RYC-H8 | - | - | + | B1 | - | - | + | B1 | ST75 |
| RYC-H7 | + | - | - | D | + | - | - | D | ST3369 |
| RYC-H28 | + | - | - | D | + | - | - | D | ST3370 |
| RYC-U29 | + | - | - | D | + | - | - | D | ST362 |
| RYC-E79 | + | - | - | D | + | - | - | D | ST925 |
| RYC-C19 | + | - | - | D | + | - | - | D | ST3374 |
| RYC-B64 | + | - | - | D | + | - | - | D | ST70 |
| RYC-C92 | + | - | - | D | + | - | - | D | ST59 |
| RYC-H33 | + | - | - | D | + | - | - | D | ST59 |
| RYC-C75 | + | - | - | D | + | - | - | D | ST59 |
| RYC-E12 | + | - | - | D | + | - | - | D | ST59 |
| RYC-U36 | + | - | - | D | + | - | - | D | ST354 |
| RYC-B69 | + | - | - | D | + | - | - | D | ST648 |
| RYC-H26 | + | - | + | D | + | - | + | D | ST648 |
| RYC-E60 | + | - | + | D | + | - | + | D | ST3368 |
| RYC-U24 | + | + | + | B2 | + | + |  | B2 | ST998 |
| RYC-T4 | + | + | - | B2 | + | + | - | B2 | ST3371 |
| RYC-C23 | + | + | + | B2 | + | + | + | B2 | ST95 |
| RYC-E76 | + | - | + | D | + | + | + | B2 | ST537 |
| RYC-U86 | + | + | + | B2 | + | + | + | B2 | ST12 |
| RYC-T5 | + | + | + | B2 | + | + | + | B2 | ST929 |
| RYC-U20 | + | + | + | B2 | + | + | + | B2 | ST73 |
| RYC-U23 | + | + | + | B2 | + | + | + | B2 | ST73 |
| RYC-H71 | + | + | + | B2 | + | + | + | B2 | ST73 |
| RYC-H72 | + | + | + | B2 | + | + | + | B2 | ST73 |
| RYC-C16 | + | + | + | B2 | + | + | + | B2 | ST73 |
| RYC-C47 | + | + | + | B2 | + | + | + | B2 | ST73 |
| RYC-U77 | + | + | + | B2 | + | + | + | B2 | ST73 |
| RYC-U43 | + | + | + | B2 | + | + | + | B2 | ST73 |
| RYC-U9 | + | + | + | B2 | + | + | + | B2 | ST73 |
| RYC-E51 | + | + | + | B2 | + | + | + | B2 | ST73 |
| RYC-E29 | + | + | + | B2 | + | + | + | B2 | ST978 |
| RYC-E40 | + | + | + | B2 | + | + | + | B2 | ST3367 |
| RYC-E42 | - | - | + | B1 | + | + | + | B2 | ST978 |
| RYC-E33 | - | - | + | B1 | + | + | + | B2 | ST3366 |
